# Effects of Pioglitazone on Nonalcoholic Fatty Liver Disease in the Absence of Constitutive Androstane Receptor Expression

**DOI:** 10.1155/2018/9568269

**Published:** 2018-09-27

**Authors:** Hwa Young Ahn, Hwan Hee Kim, Ji-Yeon Hwang, Changhun Park, Bo Youn Cho, Young Joo Park

**Affiliations:** ^1^Department of Internal Medicine, Chung-Ang University College of Medicine, 102, Heukseok-ro, Dongjak-gu, Seoul 06973, Republic of Korea; ^2^Seoul National University Hospital Biomedical Research Institute, 101, Daehak-ro, Jongno-gu, Seoul 03080, Republic of Korea; ^3^Preclinical Experimental Center, Seoul National University Bundang Hospital, 82, Gumi-ro 173 Beon-gil, Bundang-gu, Seongnam-si, Gyeonggi-do 13620, Republic of Korea; ^4^Clinical Trials Center, Severance Hospital, Yonsei University Health System, 50-1, Yonsei-ro, Seodaemun-gu, Seoul 03722, Republic of Korea; ^5^Department of Internal Medicine, Seoul National University College of Medicine, 101, Daehak-ro, Jongno-gu, Seoul 03080, Republic of Korea

## Abstract

Nonalcoholic fatty liver disease or steatohepatitis (NAFLD/NASH) is a fatty liver disease that is closely related to obesity, diabetes, and dyslipidemia. Pioglitazone, which was developed as an antidiabetic drug, is known to improve NALFD. Pioglitazone is metabolized by multiple cytochrome P450 (CYP) enzymes, which are regulated by the xenobiotic receptor constitutive androstane receptor (CAR). In this study, we investigated the effects of pioglitazone on NAFLD by absence of CAR activity under high-fat (HF)-fed conditions. CAR^−/−^ mice showed significant improvement in NALFD after 12 weeks of pioglitazone treatment compared to wild-type mice. This improvement in NAFLD persisted in CAR^−/−^ mice regardless of blood pioglitazone concentration. The expression of lipogenesis genes in the liver, sterol-regulatory element binding protein-1c (SREBP-1c), and stearoyl-CoA desaturase (SCD)-1 was decreased after pioglitazone treatment in HF-fed CAR^−/−^ mice. In addition, the expression of peroxisome proliferator-activated receptor gamma 2 (PPAR*γ*2) was decreased by pioglitazone in HF-fed CAR^−/−^ mice. Changes in SREBP-1c and PPAR *γ*2 remained constant over short-term (6 h) pioglitazone and lipid injection. Our results showed that NAFLD was improved significantly by pioglitazone in a CAR deletion state. These results might be valuable because they suggest that interaction with CAR and pioglitazone/PPAR*γ*2 may be important in regulating gene expression associated with NAFLD.

## 1. Introduction

Nonalcoholic fatty liver disease or steatohepatitis (NAFLD/NASH) is a fatty liver disease caused by diet-induced obesity. A recent epidemiologic study reported that the prevalence of NAFLD is 25.24% worldwide and 7.6% in children [1]. Obesity, type 2 diabetes mellitus, and dyslipidemia are closely related to NAFLD [2] and contribute to disease progression to liver fibrosis and cirrhosis [3]. Insulin resistance is a well-recognized risk factor for NAFLD [4]. Hyperinsulinemia resulting from insulin resistance increases not only lipid synthesis, but also fatty acid uptake by hepatocytes because of increased lipolysis of adipocytes [2]. Lifestyle intervention (diet and exercise) and pharmacological treatments, such as vitamin E, pioglitazone, and pentoxifylline, are used to treat NAFLD [3].

Among these drugs, pioglitazone was shown to improve NAFLD in some human studies [5, 6]. Pioglitazone also decreases fasting and postprandial glucose levels by improving insulin sensitivity in type 2 diabetes mellitus [7] and is currently used as an antidiabetic medication. Pioglitazone acts by binding to the peroxisome proliferator-activated receptor gamma (PPAR*γ*), a member of the nuclear receptor superfamily that plays a key role in glucose regulation and lipid metabolism [8]. Pioglitazone is extensively metabolized by hydroxylation and oxidation in the liver to form at least four primary metabolites (M-I, M-II, M-IV, and M-V) and two secondary metabolites (M-III and M-VI) [9]. Pharmacologically active M-IV and M-III are the main metabolites found in the human serum.

Pioglitazone is metabolized by multiple cytochrome P450 (CYP) enzymes, mainly CYP2C8, CYP3A4, and CYP2C9 [9, 10], which are regulated by the xenobiotic receptor constitutive androstane receptor (CAR) [11]. Previous studies showed that CAR can cause differences in drug efficacy by altering the degree of drug metabolism. For example, the acetaminophen-metabolizing enzyme CYP1A2, CYP3A11, and glutathione* S*-transferase are activated in a CAR-dependent manner after treatment with acetaminophen in wild-type mice and induced hepatotoxicity, but not in CAR null mice [12]. Therefore, CAR activity may affect the metabolism of pioglitazone and the effects of pioglitazone may vary according to the degree of metabolism.

In addition, the activity of CAR affects liver steatosis. The stimulation of CAR expression by a CAR agonist (TCPOBOP) reduced steatohepatitis in methionine choline-deficient diet-fed mice [13]. CAR is a member of the NR1 subfamily; several other nuclear receptors such as pregnane X receptor; PPAR*α*, *β*, *γ*; liver X receptors *α*, *β*; and farnesoid X receptor *α* are also members of the NR1 subfamily and are related to the pathogenesis of NAFLD [14]. Thus, interindividual differences in the effects of pioglitazone to NAFLD may be affected by CAR activity and interactions between several genes.

In this study, we hypothesized that the effect of pioglitazone on NAFLD is influenced by CAR activity. To confirm this hypothesis, we examined the effect of CAR deletion on changes in NAFLD and related gene expression induced by pioglitazone in the mouse liver.

## 2. Material and Methods

### 2.1. Animals and Study Drugs

CAR^+/+^ (wild-type, C57BL/6J) mice were supplied by Orient Bio, Inc. (Seongnam, Korea). Wild-type mice were divided into two groups (control versus CAR activation). To induce CAR activation, once per week intraperitoneal injection of 3 mg/kg of TCPOBOP (Sigma-Aldrich, St. Louis, MO, USA) was administered. Homozygous CAR knockout (CAR^−/−^) mice were generated by gene targeting as previously described [15] and then backcrossed to C57BL/6J mice to the tenth generation. They were backcrossed to CAR^+/+^ C57BL/6J mice (Orient Bio) and used as controls. Mice were housed at ambient temperature (23 ± 1°C), with 12:12-h light–dark cycles and access to water* ad libitum*.

In the normal chow diet (Purina irradiated laboratory chow 38057, Purina Korea, Seoul, Korea) and high-fat diet (60 kcal % fat diet, D12492, Research Diets, Inc., New Brunswick, NJ, USA) feeding condition, 8–10-week-old male CAR^+/+^ (control and TCPOBOP-treated) and CAR^−/−^ mice were assigned to vehicle or treatment groups according to the administration of pioglitazone hydrochloride (Takeda Chemical Industries, Osaka, Japan). Pioglitazone hydrochloride was administered at 10 mg/kg/day by the oral route by being mixed with the diet for 12 weeks. Each experimental group included at least 4 mice, and the experiment was repeated 3 times.

During administration of different concentrations of pioglitazone hydrochloride (Santa Cruz Biotechnology, Inc., Santa Cruz, CA, USA) to CAR^+/+^ and CAR^−/−^ mice (1, 3, 10, 20, or 30 mg/kg), pioglitazone was administered using sonde once daily for 14 days. Pioglitazone hydrochloride was dissolved in Solutol HS-15 (9% in phosphate-buffered saline). Similar serum concentrations of pioglitazone were detected when we administered different concentration of pioglitazone to CAR^+/+^ mice (10 and 20 mg/kg) and CAR^−/−^ mice (1 and 3 mg/kg).

To detect short-term changes of gene expression after pioglitazone treatment, we performed a short-term (6 h) experiment. Mice were divided into 4 groups of CAR^+/+^ and CAR^−/−^ mice depending on whether pioglitazone and/or lipid was administered. Three mice were included in each group. Pioglitazone hydrochloride (20 mg/kg) and 3 g/kg of 20% intralipid (LIPO MCT injection, Dongkook Pharmaceutical Co., Chungbuk, Korea) were administered via intraperitoneal injection.

All animals were sacrificed after fasting for 6 h starting from 06:00 a.m. Mice were anesthetized by intraperitoneal injection of Zoletil® (Virbac, Carros, France) and total body fat was measured by a small animal body composition analyzer, PIXImus (GE Healthcare, Little Chalfont, UK). The liver was quickly removed, weighed, and frozen in liquid nitrogen for RNA extraction. White and brown fat were also removed, weighed, and frozen in liquid nitrogen. The study protocol was approved by the Institutional Animal Care and Use Committee at the Seoul National University Bundang Hospital, Seongnam, Republic of Korea (BA1012-074/068-1).

### 2.2. Measurement of Body Weight and Glucose Tolerance

Body weight was monitored every week. Food intake was measured every 3 days. Blood glucose levels were checked with reagent strips read in a glucometer (ACCU-CHEK Active, Roche, Mannheim, Germany). An intraperitoneal glucose tolerance test was carried out after 12 h of fasting by intraperitoneal injection of 1 g/kg glucose at 12 weeks after the experiments. Blood glucose levels from tail vein blood were determined using a glucometer before and 15, 30, 60, 90, and 120 min after glucose injection.

### 2.3. Measurement of Lipid Profile and Insulin

Serum total cholesterol, triglyceride, high-density lipoprotein cholesterol, and low-density lipoprotein cholesterol were determined with a Beckman Coulter AU480 automatic biochemistry analysis system (Brea, CA, USA) with reagent kits provided by the manufacturer. For lipid extraction, tissue were rinsed with ice cold PBS to remove excess blood thoroughly and small pieces and homogenized them in 100-200*μ*L PBS with a glass homogenizer on ice. The resulting suspension was stored overnight at −20°C. To further break to cell membranes, two freeze-thaw cycles were performed. After that, the homogenates were centrifuged for 5 minutes at 5000 x *g*. The supernatant was used for assay. Triglyceride ELISA kit (Aviva Systems Biology, San Diego, CA, USA) and total cholesterol assay kit (BioVision Inc., Milpitas, CA) were used for assay. Insulin was measured using a mouse insulin ELISA kit (ALPCO Diagnostics, Windham, NH, USA) following the manufacturer's protocol.

### 2.4. Histological Analysis

The left lobes of the livers were removed, rinsed with PBS, fixed in 10% formaldehyde-PBS solution, and embedded in paraffin. Tissues were sectioned at 5 *μ*m thickness and stained with hematoxylin and eosin.

### 2.5. Measurement of Pioglitazone Concentration

The concentrations of pioglitazone were analyzed by high-performance liquid chromatography (Agilent 1200 series, Agilent Technologies, Santa Clara CA, USA). Pioglitazone hydrochloride was diluted in 50% acetonitrile to obtain a 100 *μ*g/mL working solution. This working solution was diluted with blank plasma to prepare standard solutions of different concentrations (5, 10, 50, 100, 500, 1000, and 2000 ng/mL). This standard solution (0.2 mL) was mixed with 10 *μ*L of 1 *μ*L/mL formoterol and 600 *μ*L of acetonitrile and then centrifuged for 5 min at 13,226 x *g*. Next, 100 *μ*g/mL of supernatant was mixed with 500 *μ*L of 5 mM ammonium formate: acetonitrile (20:80, 0.1% trifluoroacetic acid). This mixture (0.1 *μ*L) was subjected to liquid chromatography-mass spectrometry/mass spectrometry and the graphs were analyzed.

### 2.6. RNA Isolation and Quantitative Real-Time PCR

Total RNA was isolated from frozen liver and cells using TRIzol® Reagent (Invitrogen, Carlsbad, CA, USA) according the manufacturer's instructions. First-strand cDNA was synthesized from 1 *μ*g of RNA using an Omniscript RT kit (Qiagen, Hilden, Germany). Quantitative RT-PCR was performed using SsoFast EvaGreen Supermix (Bio-Rad Laboratories, Inc., Hercules, CA, USA) and CFX96^TM^ real-time PCR system (Bio-Rad Laboratories, Inc.). The sequences (5′ to 3′) for the primers were as follows: PPAR*α*, forward primer (AATCCTGTGCCAACCAGAAG), reverse primer (ATCGCCACTAAGGTGTCAGG); PPAR*γ*1, forward primer (TGCAGCTCAAGCTGAATCAC), reverse primer (ACGTGCTCTGTGACGATCTG); PPAR*γ*2, forward primer (TGCAGCTCAAGCTGAATCAC), reverse primer (CACGTGCTCTGTGACGATCT); peroxisome proliferator-activated receptor *γ* coactivator 1 *α* (PGC-1*α*), forward primer (AAGAGCGCCGTGTGATTTAC), reverse primer (TGCATTCCTCAATTTCACCA); fatty acid translocase, cluster of differentiation 36 (CD36), forward primer (AAACCAGTGCTCTCCCTTGA), reverse primer (CTGCACCAATAACAGCTCCA); sterol-regulatory element binding protein-1c (SREBP-1c), forward primer (TGACCCGGCTATTCCGTGA), reverse primer (CTGGGCTGAGCAATACAGTTC); fatty acid synthase (FAS), forward primer (CCCTTGATGAAGAGGGATCA), reverse primer (CAAGGCGTTAGGGTTGACAT); stearoyl-CoA desaturase (SCD)-1, forward primer (AGGTGCCTCTTAGCCACTGA), reverse primer (CCAGGAGTTTCTTGGGTTGA); carnitine palmitoyltransferase 1 (CPT-1) *α*, forward primer (ACAGTGGGACATTCCAGGAG), reverse primer (AAGGAATGCAGGTCCACATC); long-chain acyl-CoA dehydrogenase (LCAD), forward primer (TCACCACACAGAATGGGAGA), reverse primer (TTTCTCTGCGATGTTGATGC); microsomal triglyceride transfer protein (MTTP), forward primer (GTATTCCCACCTCAGCCAGA), reverse primer (GTCAGGCACGTCAAAGCATA); glyceralehyde-3-phosphate dehydrogenase (GAPDH), forward primer (TGTGTCCGTCGTGGATCT-GA), reverse primer (CCTGCTTCACCACCTTCTTGA).

### 2.7. Statistical Analysis

Statistical analysis was performed by nonparametric analysis by using the Mann–Whitney test. Statistical significance was assumed at* P* < 0.05.

## 3. Results

### 3.1. Changes in Body, Liver, and Fat Weight and Glucose and Lipid Metabolism by Pioglitazone Treatment and Different CAR Activities in Diet-Induced Obesity Mice

**Figure 1:**
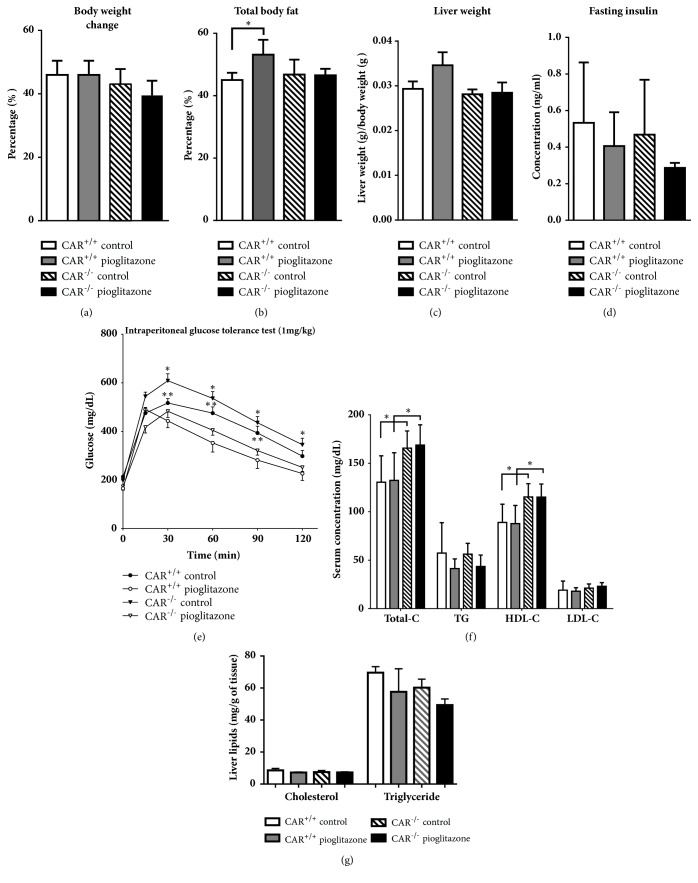
**Metabolic changes according to CAR activity. (a) Rates of change in body weight by pioglitazone treatment after 12 weeks in CAR** ^**+/+**^ **and CAR** ^**-/-**^ **mice. (b) Total body fat changes after 12 weeks. (c) Liver weight after 12 weeks. (d) Fasting insulin level after 12 weeks. (e) Intraperitoneal glucose tolerance test after 12 weeks.** *∗* p < 0.05, CAR^−/−^ control versus CAR^−/−^ pioglitazone, *∗∗* p < 0.05, CAR^+/+^ control versus CAR^+/+^ pioglitazone.** (f) Lipid profile after 12 weeks.** *∗* p < 0.05. ** (G) Measurement of cholesterol and triglyceride in the liver.**

The change in body weight in mice fed a high-fat (HF) diet for 12 weeks was not significant by pioglitazone treatment in CAR^+/+^ and CAR^−/−^ mice (Figure 1(a)). The percentage of total body fat was increased in pioglitazone-treated CAR^+/+^ mice, but this effect was reversed by CAR deletion (Figure 1(b)). Amount of food intake was similar between groups (data shown). Liver weight did not differ between groups (Figure 1(c)). Although fasting insulin level showed no significant difference (Figure 1(d)), pioglitazone treatment significantly inhibited the increase in blood glucose level at all-time points in CAR^−/−^ mice and significantly increased blood glucose in CAR^+/+^ mice group except for at 120 min after glucose loading (Figure 1(e)). Serum total and high-density lipoprotein cholesterol levels were significantly higher in CAR^−/−^ mice than in CAR^+/+^ mice. However, there was no significant change in lipid profiles after pioglitazone treatment (Figure 1(f)). Liver cholesterol and triglyceride were also not different after pioglitazone treatment (Figure 1(g)).

### 3.2. Improvement of NAFLD in Pioglitazone-Treated CAR^−/−^ Mice

**Figure 2:**
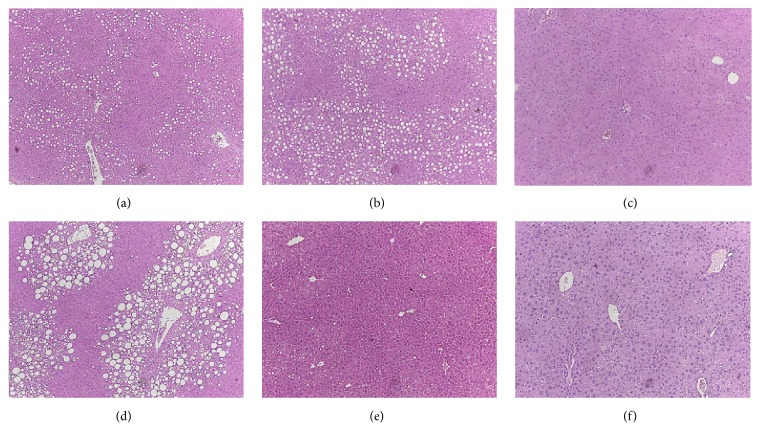
**Hepatocyte fat accumulation after 12 weeks of pioglitazone treatment according to CAR activity. Representative hematoxylin and eosin staining of liver of mice. (a) CAR** ^**+/+**^ **control. (b) CAR** ^**-/-**^ **control. (c) CAR** ^**+/+**^ **TCPOBOP. (d) CAR** ^**+/+**^ **with pioglitazone. (e) CAR** ^**-/-**^ **with pioglitazone. (f) CAR** ^**+/+**^ **TCPOBOP with pioglitazone**.

In histologic examination, hepatic steatosis was slightly aggravated in CAR^−/−^ mice (Figure 2(b)) compared with CAR^+/+^ mice (Figure 2(a)), but was nearly completely abolished after TCPOBOP treatment, which is a strong activator of CAR (Figure 2(c)). After pioglitazone treatment, fat globules remained in the liver of CAR^+/+^ mice (Figure 2(d)). In contrast, liver steatosis was markedly improved by pioglitazone treatment in CAR^−/−^ mice (Figure 2(e)). However, in TCPOBOP-treated mice, we observed no additional effect of pioglitazone on NAFLD (Figure 2(f)), possibly because the strong effects of TCPOBOP ameliorate fatty liver [16].

### 3.3. Improvement of NAFLD in Pioglitazone-Treated CAR-/- Mice Regardless of Blood Concentration of Pioglitazone

**Figure 3:**
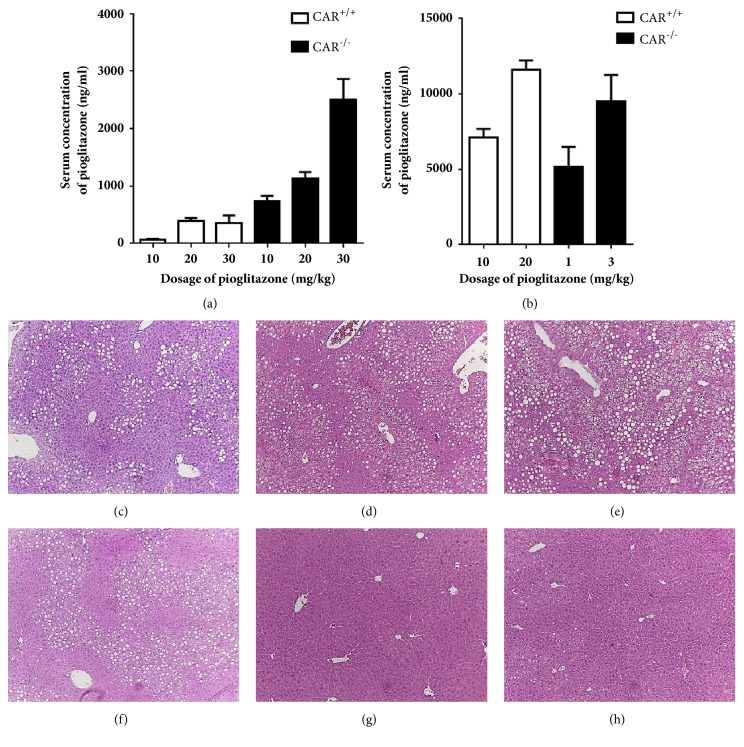
**(a, b) Serum concentrations of pioglitazone in CAR** ^**+/+**^ **and CAR** ^**-/-**^ **mice after 2 weeks of pioglitazone treatment. (c–h) Liver histology when serum concentration of pioglitazone was similar between CAR** ^**+/+**^ **and CAR** ^**-/-**^ **mice. Serum concentration was similar between (d) and (g), and (e) and (h).** (c) CAR^+/+^ control (vehicle), (d) CAR^+/+^ pioglitazone 10 mg/kg, (e) CAR^+/+^ pioglitazone 20 mg/kg, (f) CAR^−/−^ control (vehicle), (g) CAR^−/−^ pioglitazone 1 mg/kg, (h) CAR^−/−^ pioglitazone 3 mg/kg.

Because pioglitazone is mainly metabolized by CYP2C8 and CYP3A4* in vitro* [17], serum concentrations of pioglitazone may be affected by CAR activity. Therefore, we measured the concentration of pioglitazone in three groups with different CAR activity. The mean concentrations of pioglitazone were 14.9 ± 11.5, 2054.0 ± 1132.9, and 4109.7 ± 606.2 ng/mL in CAR^+/+^ with TCPOBOP, CAR^+/+^, and CAR^−/−^ mice, respectively. Because TCPOBOP strongly stimulates CAR activity, we could not properly evaluate the effect of pioglitazone under TCPOBOP treatment conditions. In addition, as pioglitazone induced expression of CAR target genes (CYP2B10 and CYP3A11, Supplementary Figure 1), we thought that the existence of CAR gene itself might interfere with the interpretation of the effect of pioglitazone on NAFLD. Thus, further analysis of the effects of pioglitazone on NAFLD by CAR activity was performed only in CAR^+/+^ and CAR^−/−^ mice. To confirm whether the improvement of hepatic steatosis after pioglitazone in CAR^−/−^ mice resulted from a higher concentration of pioglitazone or was related to the absence of CAR itself, we made similar serum pioglitazone concentrations for both CAR^+/+^ and CAR^−/−^ mice. We administered different concentrations of pioglitazone (10, 20, and 30 mg/kg) for 2 weeks with the HF diet to CAR^+/+^ and CAR^−/−^ mice and measured the pioglitazone concentration in the blood (Figure 3(a)). CAR^−/−^ mice showed approximately 3–10-fold higher concentrations of pioglitazone compared to CAR^+/+^ mice after receiving the same dose of pioglitazone. Based on these results, we administered 10 and 20 mg/kg of pioglitazone in CAR^+/+^ mice and 1 and 3 mg/kg of pioglitazone to CAR^−/−^ mice for 2 weeks in conjunction with a HF diet. We obtained similar serum concentrations of pioglitazone in CAR^+/+^ and CAR^−/−^ mice (Figure 3(b)). Next, we compared the hepatic steatosis of CAR^−/−^ mice treated with 1 and 3 mg/kg of pioglitazone to that in CAR^+/+^ mice treated with 10 and 20 mg/kg of pioglitazone. Despite the similar serum concentrations of pioglitazone between CAR^+/+^ and CAR^−/−^ mice, a significant improvement in hepatic steatosis was persistently observed in CAR^−/−^ mice (Figure 3(d) versus 3(g), 3(e) versus 3(h)). In CAR^+/+^ mice, pioglitazone treatment showed no improvement in fatty liver compared with control mice (Figures 3(d), 3(e) versus Figure 3(c)). On the other hand, pioglitazone treatment was effective in the improvement of fatty liver in CAR^−/−^ mice compared to the control mice (Figures 3(g), 3(h) versus 3(f)).

### 3.4. Changes in Gene Expression Related to Hepatic Steatosis in 12 Weeks of HF Diet Feeding to CAR^−/−^ Mice after Pioglitazone Treatment

**Figure 4:**
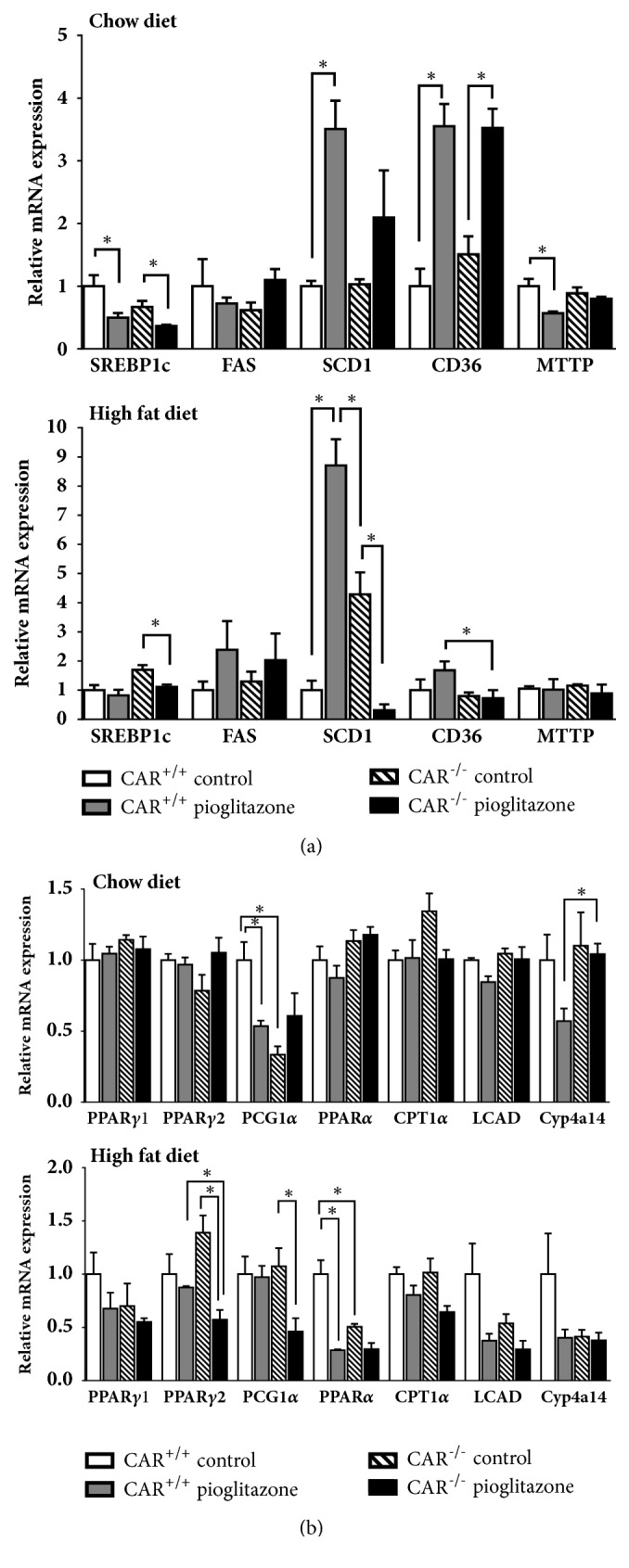
**Gene expression related to fatty liver of CAR** ^**+/+**^ **and CAR** ^**-/-**^ **mice after 12 weeks of pioglitazone treatment in chow and high-fat diet condition. (a) Difference in expression of genes involved in lipogenesis, SREBP-1c, FAS, and SCD-1, and lipid uptake, CD36 and MTTP. (b) Difference in expression of PPARs and PGC1*α* and genes involved in fatty acid oxidation, CPT1*α*, LCAD, and CYP4A14**. *∗*p < 0.05. CD36, cluster of differentiation 36; CPT1*α*, carnitine palmitoyltransferase 1*α*; CYP4A14, cytochrome P450, family 4, subfamily a, polypeptide 14; FAS, fatty acid synthase; LCAD, long-chain acyl-CoA dehydrogenase; MTTP, microsomal triglyceride transfer protein; PGC1*α*, peroxisome proliferator-activated receptor *γ* coactivator 1*α*; PPAR, peroxisome proliferator-activated receptor; SREBP-1c, sterol-regulatory element binding protein-1c; SCD-1, stearoyl-CoA desaturase.

To investigate whether the mechanism among those related to the development of fatty liver was associated with the improvement in hepatic steatosis observed in HF-fed CAR^−/−^ mice with pioglitazone treatment, we analyzed changes in the mRNA expression of several well-known genes related to lipogenesis, hepatic lipid uptake, and fatty acid oxidation according to diet, pioglitazone, and CAR status (Figure 4). Expression of SREBP1c involved in lipogenesis was decreased after pioglitazone treatment in both chow and HF-fed CAR^−/−^ mice (Figure 4(a)). These decreases by pioglitazone were not observed in HF-fed CAR^+/+^ mice. Particularly, SCD-1 expression was increased in pioglitazone-treated CAR^+/+^ mice, but significantly decreased in HF-fed pioglitazone-treated CAR^−/−^ mice. In addition, the expression of CD36, which is important for fatty acid uptake, was increased after pioglitazone treatment in the chow diet, but the increase was not observed in HF-fed CAR^−/−^ mice (Figure 4(a)). Because both SCD-1 and CD36 were regulated by PPAR*γ*, the expression of PPAR*γ*1, PPAR*γ*2, and PGC1*α* was evaluated; interestingly, the expression of PPAR*γ*2 and PGC1*α* was decreased only in HF-fed pioglitazone-treated CAR^−/−^ mice (Figure 4(b)). The expression of fatty acid oxidation-related genes did not differ among HF-fed CAR^+/+^ and CAR^−/−^ mice, except PPAR*α*; the expression of PPAR*α* was decreased by pioglitazone or CAR deletion under HF-fed conditions (Figure 4(b)).

### 3.5. Acute Changes in Gene Expression after Pioglitazone Treatment in CAR^−/−^ Mice

**Figure 5:**
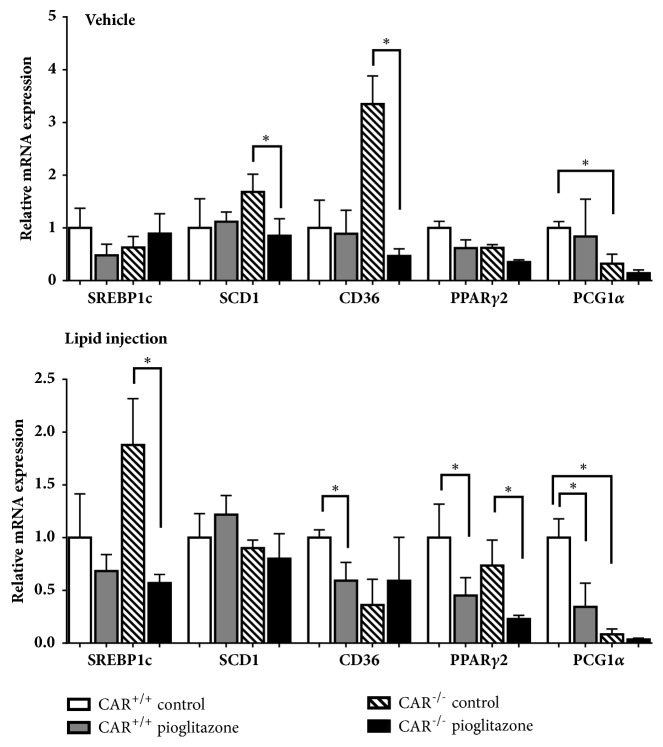
**Expression of five genes (SREBP-1c, SCD-1, CD36, PPAR*γ*2, and PGC1*α*) in CAR** ^**+/+**^ **and CAR** ^**-/-**^ **mice after 6 h pioglitazone and lipid administration via intraperitoneal injection.** *∗* p < 0.05. CD36, cluster of differentiation 36; PGC1*α*, peroxisome proliferator-activated receptor *γ* coactivator 1*α*; PPAR*γ*2, peroxisome proliferator-activated receptor *γ*2; SREBP-1c, sterol-regulatory element binding protein-1c; SCD-1, stearoyl-CoA desaturase.

To determine whether the changes in gene expression were primary or secondary responses after long-term of treatment with the HF diet or pioglitazone, we analyzed the expression of SREBP-1c, SCD-1, CD36, PPAR*γ*2, and PCG1*α* at 6 h after pioglitazone treatment combined with vehicle or lipid injection (Figure 5). The expression of SREBP-1c was increased in CAR^−/−^ mice and significantly decreased by pioglitazone combined with lipid injection. This change was like that observed in 12-week HF-fed mice. The expression of SCD-1 and CD36 was decreased in pioglitazone-treated CAR^−/−^ mice with vehicle injection, but this change was not observed in CAR^−/−^ mice with lipid injection. PPAR*γ*2 expression was significantly decreased by pioglitazone in both CAR^+/+^ and CAR^−/−^ mice with lipid injection. The expression of PGC1*α* was decreased in CAR^−/−^ mice, regardless of lipid injection.

## 4. Discussion

In this study, we evaluated the effect of pioglitazone on NAFLD mediated by CAR deletion in the mouse liver. Our results showed that NAFLD was significantly improved by pioglitazone in a CAR deletion state. This effect was not due to the elevated serum concentration of pioglitazone resulting from decreased degradation by CAR deletion.

CAR agonism has been shown to improve fatty liver and insulin resistance [18]. In contrast, in the absence of CAR, there was no improvement in fatty liver, as shown in Figure 2(d), and these results were confirmed in another study [16]. Therefore, it is likely that the improvement of fatty liver in pioglitazone-treated CARKO mice is not due to CAR deletion itself but rather because of the interaction of several genes involved in fatty liver in the absence of CAR.

Genetic analysis revealed that CD36 and SCD-1 expression was significantly decreased by pioglitazone in 12-week HF-fed CAR^−/−^ mice and these changes may have contributed to the removal of liver fat. Recent findings suggested that increased hepatic CD36 activity is critical for the development of steatosis under pathologic conditions such as HF diet, obesity, and diabetes [19, 20]. In contrast, when CD36 was deleted, the liver was protected from NAFLD development [21]. SCD-1 is an endoplasmic reticulum enzyme that catalyzes the biosynthesis of monounsaturated fatty acids from saturated fatty acids [22]. Hepatic SCD-1 activity is increased in NAFLD [23]. Mice with liver-specific knockout of SCD1 are protected from carbohydrate-induced adiposity and hepatic steatosis [24]. These results suggest that deceased expression of CD36 and SCD-1 protects against hepatic steatosis. However, the responses of SCD-1 and CD36 differed in 12-week (long-term) and 6-h (short-term) administration of pioglitazone in CAR^−/−^ mice. Therefore, the changes in SCD-1 and CD36 are likely a secondary response.

In contrast, the expression of SREBP-1c in CAR^−/−^ mice fed a HF diet was consistently decreased by pioglitazone in both the 12-week and 6-h experiments, suggesting that decreased expression of SREBP-1c has a primary effect on the reduction of hepatic steatosis. In addition, the change in the pattern of PPAR*γ*2 was similar to that in SREBP-1c, suggesting that these genes are closely related, which has been demonstrated previously [25]. We also found that HF diet itself increases the expression of SREBP1c and PPAR*γ*2 in the liver (Supplementary Figure 2). Therefore, the decreased expression of SREBP-1c and PPAR*γ*2 by pioglitazone in HF-fed CAR^−/−^ mice played a major role in improving hepatic steatosis.

Several studies examining the effects of PPAR*γ* on NAFLD have demonstrated enhanced expression of lipogenic genes and increased expression of PPAR*γ* in animal models of steatotic liver [26–28]. Moreover, a role for PPAR*γ* has been established in the maintenance of a steatotic phenotype in the liver [27]. In a previous study using a hepatic stable cell line expressing PPAR*γ*2, PPAR*γ*2 expression induced lipid accumulation in hepatocytes by upregulating adipogenic and lipogenic gene expression [29]. Culturing of PPAR*γ*2-expressing hepatocytes in the absence of serum (exogenous lipids) resulted in lipid accumulation, suggesting that de novo lipid synthesis is an important mechanism contributing to steatosis in hepatocytes [29].

Interestingly, pioglitazone did not improve fatty liver in HF-fed CAR^+/+^ mice, despite improvements in blood glucose level. This effect was also demonstrated in other murine studies. C57BL6 ob/ob mice treated with rosiglitazone (1 mg/kg) for 12 weeks showed significantly increased hepatic steatosis and the NAFLD activity score was significantly higher in rosiglitazone-treated mice than in untreated ob/ob mice [30]. Oral administration of pioglitazone for 28 days also worsened hepatic steatosis in KKAy mice [31]. In addition, PPAR*γ*1 overexpression in the liver of PPAR*α* ^−/−^ mice showed increased fat accumulation in the liver [28]. These results suggest that the effects of PPAR*γ* agonist on fatty liver may differ in humans and mice.

A limitation of this study was that we were not able to determine the exact molecular mechanism of the interaction between PPAR*γ*2 and CAR. A summary figure of our result is presented in Supplementary Figure 3. Further studies are needed to reveal the underlying mechanisms. However, our study results might be valuable because they suggest that interaction with CAR and pioglitazone/PPAR*γ*2 may be important in regulating gene expression associated with NAFLD.

## Data Availability

The data used to support the findings of this study are included within the article.

## Supplementary Materials

Supplementary Materials Supplementary Figure 1: the expression of CYP2B10 and CYP3A11 after 12 weeks of pioglitazone treatments in CAR^+/+^ mice with HF diet. *∗* p < 0.05. Supplementary Figure 2: difference of expression of genes (SREBP1c, SCD-1, CD36, and PPAR *γ*2) by HF diet in CAR^+/+^ mice. *∗* p < 0.05. Supplementary Figure 3: summary figure of improvement of fatty liver by interaction of PPAR *γ*2, SREBP1c, and CAR.
