# Supplementary figures and images for: Effects of Pioglitazone on Nonalcoholic Fatty Liver Disease in the Absence of Constitutive Androstane Receptor Expression
Source: PPAR Res. 2018 Sep 27;2018:9568269. doi: 10.1155/2018/9568269 (PMC6181001; doi:10.1155/2018/9568269)

Supplementary Figure 1

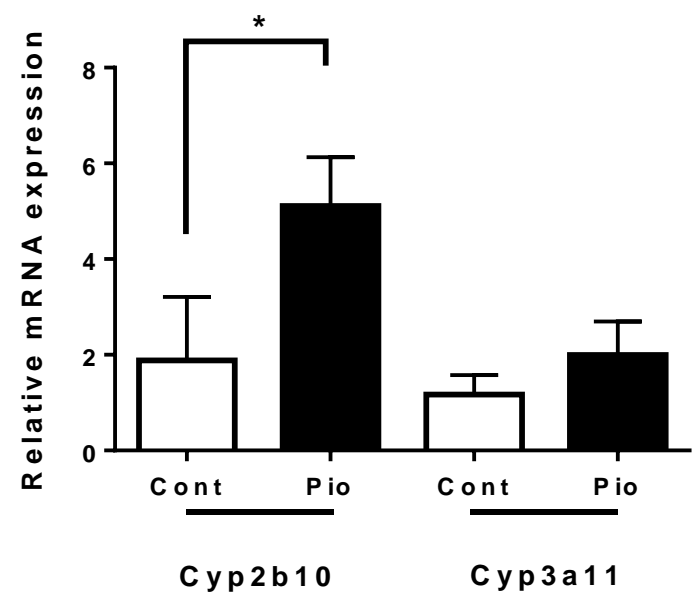

Supplementary Figure 2

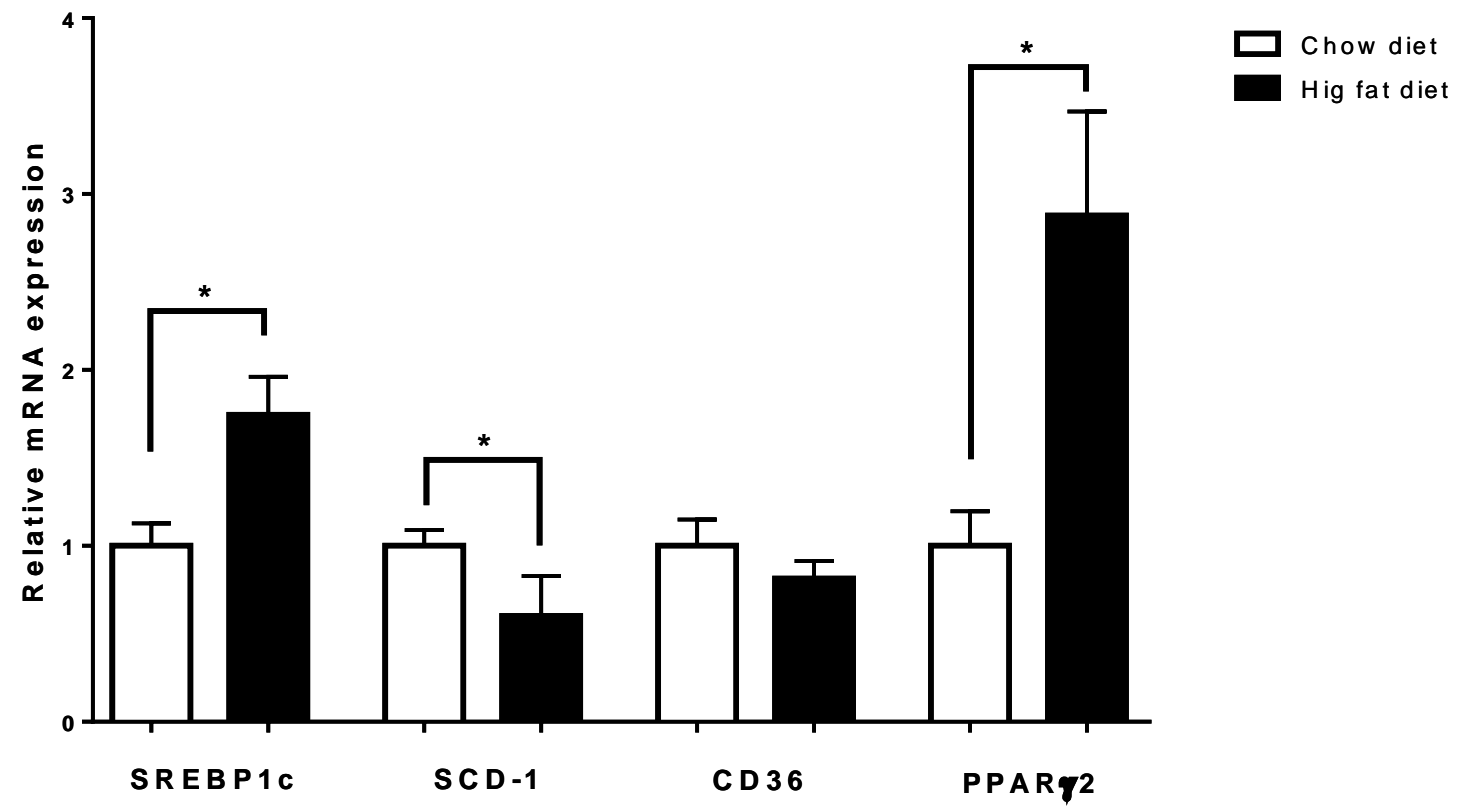

Supplementary Figure 3

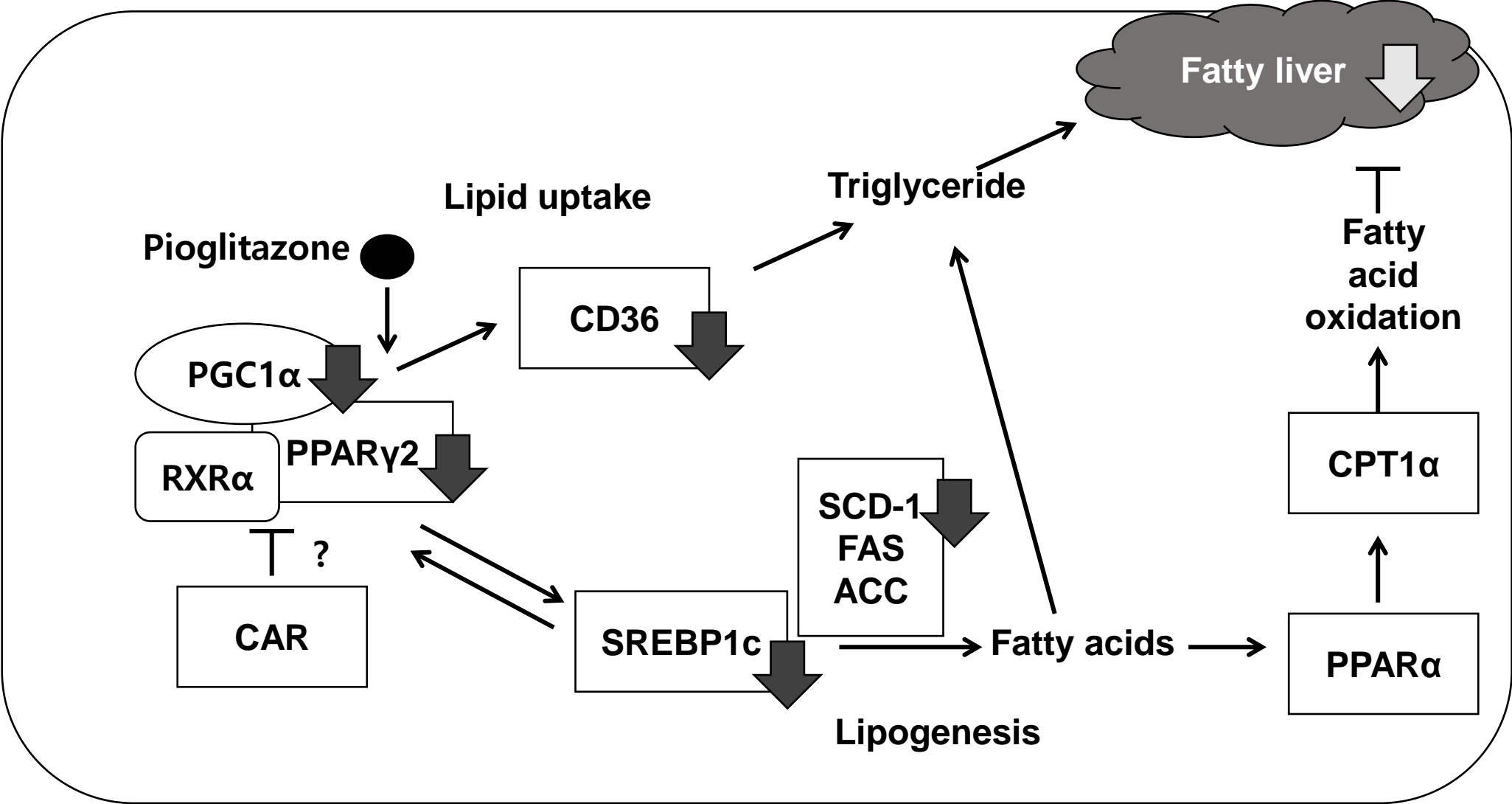

Supplement: Supplementary Materials — Supplementary Figure 1: the expression of CYP2B10 and CYP3A11 after 12 weeks of pioglitazone treatments in CAR+/+ mice with HF diet. ∗ p < 0.05. Supplementary Figure 2: difference of expression of genes (SREBP1c, SCD-1, CD36, and PPAR γ2) by HF diet in CAR+/+ mice. ∗ p < 0.05. Supplementary Figure 3: summary figure of improvement of fatty liver by interaction of PPAR γ2, SREBP1c, and CAR. [file 9568269.f1.pdf]
